# Molecular modeling of the reductase domain to elucidate the reaction mechanism of reduction of peptidyl thioester into its corresponding alcohol in non-ribosomal peptide synthetases

**DOI:** 10.1186/1472-6807-10-1

**Published:** 2010-01-12

**Authors:** Balachandran Manavalan, Senthil K Murugapiran, Gwang Lee, Sangdun Choi

**Affiliations:** 1 Department of Molecular Science and Technology, Ajou University, Suwon, 443-749, Korea; 2 Institute for Medical Sciences, Ajou University School of Medicine, Suwon, 443-721, Korea

## Abstract

**Background:**

Nonribosomal peptide synthetases (NRPSs) are multienzymatic, multidomain megasynthases involved in the biosynthesis of pharmaceutically important nonribosomal peptides. The peptaibol synthetase from *Trichoderma virens *(TPS) is an important member of the NRPS family that exhibits antifungal properties. The majority of the NRPSs terminate peptide synthesis with the thioesterase (TE) domain, which either hydrolyzes the thioester linkage, releasing the free peptic acid, or catalyzes the intramolecular macrocyclization to produce a macrolactone product. TPS is an important NRPS that does not encompass a TE domain, but rather a reductase domain (R domain) to release the mature peptide product reductively with the aid of a NADPH cofactor. However, the catalytic mechanism of the reductase domain has not yet been elucidated.

**Results:**

We present here a three-dimensional (3D) model of the reductase domain based on the crystal structure of vestitone reductase (VR). VR belongs to the short-chain dehydrogenase/reductase (SDR) superfamily and is responsible for the nicotinamide dinucleotide phosphate (NADPH)-dependent reduction of the substrate into its corresponding secondary alcohol product. The binding sites of the probable linear substrates, alamethicin, trichotoxin, antiamoebin I, chrysopermin C and gramicidin, were identified within the modeled R domain using multiple docking approaches. The docking results of the ligand in the active site of the R domain showed that reductase side chains have a high affinity towards ligand binding, while the thioester oxygen of each substrate forms a hydrogen bond with the OH group of Tyr176 and the thiol group of the substrate is closer to the Glu220. The modeling and docking studies revealed the reaction mechanism of reduction of thioester into a primary alcohol.

**Conclusion:**

Peptaibol biosynthesis incorporates a single R domain, which appears to catalyze the four-electron reduction reaction of a peptidyl carrier protein (PCP)-bound peptide to its corresponding primary alcohol. Analysis of R domains present in the non-redundant (nr) database of the NCBI showed that the R domain always resides in the last NRPS module and is involved in either a two or four-electron reduction reaction.

## Background

Nonribosomal peptide synthetases (NRPSs) are multi-enzymatic, multi-domain megasynthases that are involved in the synthesis of a remarkable array of commercially important nonribosomal peptides through the sequential condensation of amino acid monomers [1-4]. *Trichoderma virens *peptaibol synthetases (TPS) are important NRPS members that synthesize peptaibols, which are a family of short chain length peptides (≤20 residues) that harbor a unique C-terminal alcoholic group instead of a carboxyl group. Peptaibols form right-handed alpha-helical structures that self-associate into multimeric transmembrane channels. These channels conduct ionic species to disrupt the osmotic balance and promote cell death [5]. Peptaibols may also induce resistance to pathogens in plants. For example, exogenous peptaibol application activates a defense reaction in lima beans and reduces the susceptibility of tobacco to tobacco mosaic virus [6].

Peptaibol is synthesized by the TPS assembly line complex, which is a nonribosomal peptide synthase/polyketide synthase (NRPS/PKS) hybrid system. Various hybrid NRPS/PKS systems such as rapamycin, bleomycin, yersiniabactin and epothilone have already been reported in the literature [7,8]. In TPSs that are composed of one PKS and eighteen NRPS modules, PKSs always reside at the N-terminal end and initiate the reaction [9]. PKSs possess three domains, β-ketoacyl-ACP synthase (KS), acyl transferase (AT) and acyl carrier protein (ACP). In general, the KS domain catalyzes the chain extension, the AT domain is responsible for loading the starter and the ACP domain holds the growing macrolide, which is subsequently transferred to the next module. NRPS modules are iterative, with one module for one amino acid to build a peptide product. Each module required for the addition of a single monomer consists of a condensation domain (C), an adenylation domain (A) and a peptidyl carrier protein (PCP, also denoted as the thiolation domain (T)). The A domain selects the amino acid and activates it as an aminoacyl adenylate, after which it is transferred into the -SH group of the 4'-phosphopantetheinyl arm attached to the PCP domain [10]. The C domain, which is present between every consecutive pair of the A domain and the PCP, catalyzes peptide bond formation between the upstream peptidyl-S-PCP and the free amino group of the downstream aminoacyl-S-PCP, thus facilitating the translocation of the growing chain into the next module [11-13].

During biosynthesis, the growing chain remains covalently attached to the enzyme until it reaches its full length, at which point the TE domain residing at the last module releases a cyclic, branched-cyclic or linear product [10,14]. An alternative termination scheme involves reducing the tethered C-terminal residue by the R domain at the end of a NRPS module, which results in the release of a peptide with an alcoholic C-terminal [15]. Such a reductase-mediated terminal modification occurs in TPS. Wiest *et al. *[9] already proposed that the TPS C-terminal end dehydrogenase domain plays a key role in the reduction process of PCP-bound peptide to generate a primary alcohol. However, the exact mechanism by which the R domain releases the primary alcohol product has not been explored.

**Figure 1:**
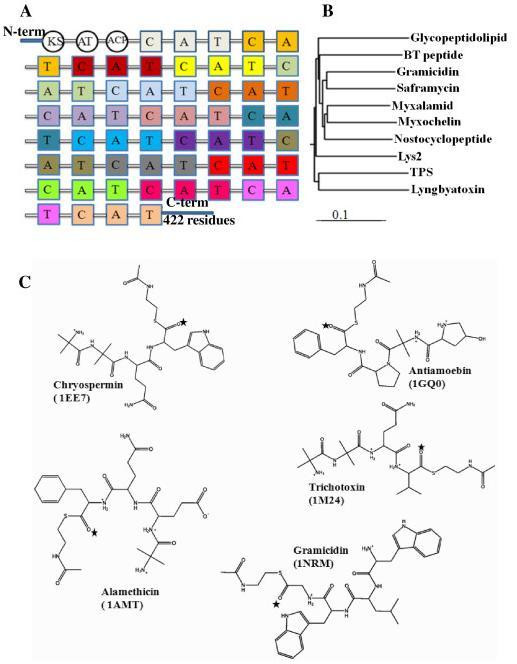
**BLAST analysis**. (A) Domain organization of TPS: Different colors depict discrete modules. Each module contains three catalytic domains shown in the same color, as well as the C-terminal containing 422 amino acids, which were investigated in detail; A: Adenylation domain; C: Condensation domain; T: Thiolation domain; KS: Ketoacyl synthase; AT: Acyl transferase; ACP: Acyl carrier protein. (B) Phylogenetic tree of the characterized NRPS proteins: The neighbor-joining algorithm was used to infer the topology based on multiple sequence alignment and Poisson distances. Bootstrap scores of >50% are presented. The accession numbers of the synthases involved in the biosynthesis of the following products were as follows: saframycin (AAC44129), lyngbyatoxin (AAT12283), myxalamid (AAK57184), myxochelin (AAG31130), gramicidin (Q70LM4), Lys2 (AAA34747), glycopeptidolipid (CAB55600), BT peptide (AAY29583), nostocyclopeptide (AA023334), and TPS (AAM78457). (C) MOE 2008.10-generated 2D ligands of the final four residues were used in reductase model docking; * represents the thioester group involved in the reduction reaction.

In this study, we identified the location of the R domain that catalyzes reduction of the PCP-bound peptide to the corresponding primary alcohol in the 422 amino acid C-terminal linker region of TPS (Figure 1A). To elucidate the function of the R domain, we initially identified similar domains present in the synthases involved in the biosynthesis of myxalamid, myxochelin, glycopeptidolipid, lyngbyatoxin and *Brevibacillus texasporus *(BT) by using BLAST searches of the nr database. The primary sequences of R domains from TPS and glycopeptidolipid show 60% sequence similarity. In order to determine the reduction reaction catalyzed by the R domain, which is common for the above synthase families, we chose glycopeptidolipid having 20.5% sequence identity with VR. Our modeling and refinement approach to explain the reduction reaction mechanism is similar to the approach used by Zhou *et al. *[16], who modeled a 3D structure of heparanase that had only 20% sequence identity with its template xylanase (1BG4). They subsequently conducted molecular dynamics (MD) refinement and tested the inhibitors targeting the heparanase model. We constructed a 3D model of the R domain based on vestitone reductase, which is a member of the SDR family for which the 3D structure has been solved by X-ray crystallography. The refined structure of the R domain was employed in the docking simulation. Analysis revealed that Tyr176 and Glu220 are the prime proton donors for the reduction of thioester into the primary alcohol via an intermediate aldehyde. Additionally, the reaction mechanism of the R domain was clearly described herein.

## Methods

### Sequence analysis and domain prediction of TPS

The TPS protein sequence extracted from the NCBI protein database (accession no. AAM78457) was submitted to NRPS-PKS, which is a database that predicts the domain organization of nonribosomal peptide synthetases and polyketide synthetases [17]. This program predicted all possible domains, including A, C, T, ACP, KS, AT, and a 422 amino acid C-terminal linker that are present in the TPS sequence. We believed that the 422-amino acid C-terminal linker was the putative R domain; therefore, we analyzed the sequence using the Conserved Domain Database (CDD) search program at NCBI [18], the Simple Modular Architecture Research Tool (SMART) search program at EMBL [19] and the Pfam search program [20]. A BLASTP search of the TPS C-terminal linker against the nr database [21] was conducted to identify previously characterized R domains present in the multidomain proteins. Additionally, we inferred the phylogenetic tree of the ten characterized proteins using the neighbor-joining method based on their CLUSTALX multiple sequence alignment [22].

### Threading analysis of characterized reductase sequences using fold recognition servers

A search was conducted to identify the possible folds for the R domain (R domain from TPS and similar characterized multidomain proteins containing the R domain) by the threading and fold recognition servers, GenTHREADER http://www.psipred.net/psiform.html[23] and PHYRE [24]. We opted for this approach because we could not obtain any relevant template structures or domains through the Pfam, SMART CDD, or BLASTP searches against the PDB. Threading analysis revealed that the folds of the R domains belong to the class extended SDR proteins family. The similarities among the folds were verified with a combinatorial extension (CE) program http://cl.sdsc.edu/[25]. The curated sequences of the R domains were taken from the GenTHREADER and PHYRE Programs, and multiple sequence alignment was performed using Tcoffee http://www.tcoffee.org/ to identify the conserved regions of the motif among these characterized proteins.

### Homology modeling and its assessment

The glycopeptidolipid R domain FASTA sequence and the crystal structure coordinates of the VR (2p4h chain X) [26] were loaded into the Molecular Operating Environment (MOE) 2008.10. The primary structure of the 2p4h and R domain were aligned and carefully checked to avoid deletions or insertions in conserved regions and corrected manually wherever necessary. A series of 10 R domain models were independently constructed with the MOE using the Boltzmann-weighted randomized procedure [27] combined with specialized logic for the handling of sequence insertions and deletions [28]. There was no difference in the number and organization of secondary structural elements and no significant main chain deviation among the 10 models generated for each R domain. However, the model with the highest packing score was selected for full energy minimization (MOE packing score = -2.2612). The overall geometric and stereo-chemical qualities of the final modeled structure of the R domain were examined using Ramachandran plots generated within the MOE and Verify 3D server [29,30]. The structural stability was evaluated by a MD simulation, while the reliability of the model was further evaluated by docking simulation and binding analysis.

### Structural refinement and stability evaluation by MD

MD simulations were conducted using GROMACS 3.3.3 [31]. The R domain structure was inserted into a cubic box maintaining a minimum of 10 Å between the box edges and the protein surface. The resulting system was solvated with simple point charge (SPC) water molecules and then minimized with the GROMOS96 force field using the steepest descent method [32]. Counter ions were added to neutralize the system. The temperature of the bath was set to 300 K and the coupling time constant was set to 0.1 ps [33]. The box pressure was maintained at 1 bar using 1 ps time constant and a water compressibility of 4.5 × 10^-5 ^bar^-1^. A cut off radius of 1 nm was used in the simulation for non-bonded interactions. After equilibrating the system, a 2-nanosecond (ns) production simulation was conducted with a 1 femtosecond (fs) time step at a pressure of 1 bar and a temperature of 300 K.

### Binding site selection and exploration

The Site Finder module of MOE 2008.10 was used to identify the possible substrate binding pockets within the newly generated 3D structure of the R domain. Hydrophobic or hydrophilic alpha spheres served as probes denoting zones of tight atom packing. These alpha spheres were used as centroids for the creation of dummy atoms used to define potential sites throughout the docking process [34,35].

### Reductase domain-ligand docking

The reductase substrates included linear peptides such as alamethicin (1AMT), antiamoebin I (1GQ0), trichotoxin (1M24), chryospermin C (1EE7) and gramicidin (1NRM) [36-39]. The above substrates are ≈18-20 amino acids long and linear. For each substrate, we took three, four (Figure 1C), five and six residues from the C-terminal end of the above crystal structure because only the final C-terminal residue is involved in the catalytic mechanism. In all of the ligand structures, we replaced the C-terminal alcohol group with C(= O)SNAc to smooth the protein-ligand docking process. It should be noted that C(= O)SNAc (peptidyl-S-N-acetylcysteamine) mimics the 4'-phosphopantetheine moiety of the PCP loaded with a substrate and has been used successfully to characterize NRPS domains [40]. Partial charges and hydrogens were added to protonated and unprotonated molecules using Merck Molecular Force Field 94× (MMFF94X), which is suitable for small drug-like molecules [41]. All of the structures were energy minimized using the conjugated gradient with the convergence criterion = 0.05 kcal/mol, ε = 1. We used these ligands for docking of the R domain using the two different docking programs described below.

### (i) MOE-DOCK 2008.10

A binding region is identified by a cluster of hydrophobic and hydrophilic alpha spheres; each of which marks their own environments. Ligand atoms are matched to the corresponding alpha spheres during the docking process. The alpha spheres are used to calculate the shape complementarity of small molecules fitting into macromolecules, as well as the binding affinities of the conformers. The above method may generate bound conformations that approach crystallographic resolutions [42]. The ligand explores the conformational space to locate the most favorable binding orientation and conformation by aligning and matching all triangles of the template points with compatible geometry, while the protein atoms remain fixed. For each ligand, 100 poses were generated and scored in an effort to determine the favorable binding modes. An affinity scoring function, ΔG, was employed to rank candidate poses. In addition, we employed the triangle matcher method that generates poses in a systematic manner and more accurate way than the alpha triangle method by aligning the ligand triplet of atoms with the triplet of alpha spheres in cavities of tight atomic packing. The docking process accounted for the thioester carboxyl groups of ligands. Poses from the molecular database for each ligand were scored based on complementarity with binding pocket alpha spheres.

### (ii) ASEDock

Alpha Sphere based protein-ligand Docking (ASEDock) is a novel fast-docking program written in scientific vector language (SVL) (MOE platform) and based on the alpha shape method. Ligand atoms have alpha spheres within 1 Å. Based on this property, concave models can be created and ligand atoms from a large number of conformations generated by superimposition with these points can be evaluated and scored by maximum overlap with alpha spheres and minimum overlap with the receptor atoms. The ligand conformations were subjected to energy minimization using the MMF94S force field [41] and when converged, reproduced the experimentally bioactive conformations. The scoring function used by ASEDock is based on protein-ligand interaction energies. The docking process took into account the thioester group of the substrate. Poses from the molecular databases for each ligand were ranked based on U_total_. For each ligand, 500 conformations were generated using the default systematic search parameters in the ASEDock module. Five thousand poses per conformation were randomly placed onto the alpha spheres located within the large receptor site. From the resulting 500,000 poses, the 200 poses with the lowest U_total _values were selected for further optimization with the MMF94S force field. During the refinement step, the ligand was free to move within the rigid binding pocket.

### Validation of the R domain ligand docking via VR-vestitone docking

The two docking methods described above were used to assess the validity of the docking predictions based on the R domain-linear substrates (alamethicin, antiamoebin I, trichotoxin, chryospermin and gramicidin) by calculating possible bound conformations of the vestitone-VR. The crystal structure of VR (2P4H, devoid of ligand) was retrieved from PDB and the vestitone ligand was downloaded from PubChem for docking. Vestitone-VR docking poses were obtained using MOE-Dock 2008.10 and ASEDock and then compared with the original crystal structure reports.

## Results

### BLAST analysis of the C-terminal linker

**Figure 2:**
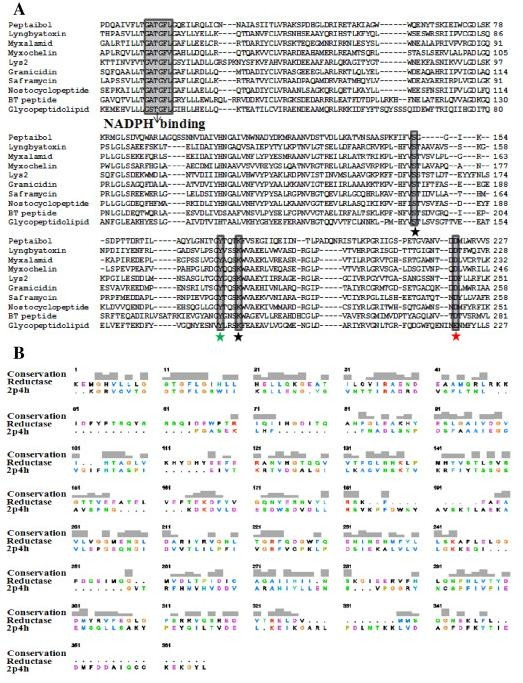
**Homology modeling of the R domain**. (A) Multiple sequence alignment of the R domains within characterized proteins: Multiple sequence alignments of the R domains from the experimentally characterized NRPS/PKS clusters. Sequence information of the synsthases can be found at Figure 1B. The alignment showed that the NADPH binding site and the catalytic site (marked with black and green asterisks) are conserved. (B) Sequence alignment used to build the R domain model (glycopeptidolipid) based on the VR template retrieved by MOE. Gray blocks indicate the level of sequence similarity. Tallest blocks: residues are identical at that position. Intermediate blocks: residues are not identical but relatively similar based on their properties. Small blocks: residues are somewhat conserved with respect to structure or function. The absence of blocks indicates no appreciable structure/function conservation. Gaps in one sequence relative to the other are indicated by dashes. The UCSF chimera visualization system was used to generate this figure [64].

The NRPS-PKS search predicted the following domains for TPS: 18 A, 18 C, 18 T, KS, AT, ACP, and a 422 amino-acid-long C-terminal linker (Figure 1A). A BLAST analysis of this C-terminal linker region revealed a high similarity to several NRPS reductases from different sources, as well as other proteins such as male sterility protein and NADPH-dependent reductases. Among 1000 hits (proteins) obtained from the BLAST analysis, ten (including our query, peptaibol) were previously characterized, well-known R domains. While the synthases that are involved in the biosynthesis of myxalamid, myxochelin A, BT peptide, lyngbyatoxin, peptaibols, glycopeptidolipid and gramicidin have been experimentally shown to reduce their substrates to corresponding alcohol via a four-electron reduction reaction [9,15,40,43-48], nostocyclopeptide, lys2 and saframycin are known to reduce their substrates to corresponding aldehyde via a two-electron reduction [49-51]. By using the neighbor-joining method, we inferred the phylogeny between these characterized proteins. Figure 1B shows the cladogram, which suggested that the TPS sequence is most closely related to the characterized proteins. Additionally, all of these proteins show the highly conserved GXXGXXG motif for NADPH binding and the SYK catalytic triad, which is represented by black and green asterisks (Figure 2A).

### Threading analysis of the R domain

The lack of a crystal structure for any R domain from the NRPS biosynthetic pathway and the high degree of sequence divergence in this family prompted us to use threading and fold recognition approaches. The GenTHREADER and PHYRE fold recognition prediction servers were used for threading analysis because they have the potential to reveal structural similarities in the absence of a high degree of similarity among sequences. All of the C-terminal linkers of the characterized proteins with differing lengths that were identified by the NRPS-PKS program were analyzed using both fold recognition servers (Table 1). The fold prediction hits with the highest level of statistical significance corresponding to p-values lower than 0.0001 were labeled as CERTAIN by the GenTHREADER server, while hits having p-values between 0.0001 and 0.001 were labeled as HIGH. We considered only those matches labeled as CERTAIN or HIGH by GenTHREADER or having a precision of 100% in the case of fold prediction by PHYRE. All 10 sequences matched the structure of SDR proteins in the PDB. These findings demonstrate that the R domain present in the NRPS protein would adopt a fold similar to that of the SDR family members in other organisms. The structures were aligned consistently with all of the sequences by both servers and had a maximum sequence identity and alignment with the query sequences. Such high sequence identities were chosen as structural templates for the R domains of the NRPS proteins. The structure of the VR (PDB code 2p4h; 310 residues) and a dTDP-glucose 4,6-dehydratase (PDB code 1r6d; 322 residues) showed alignment with most R domains. However, considering the alignment length and length of the query sequence, 2p4h was selected as a template for the R domain for further analysis. These results suggest that the R domains present in NRPS proteins are likely to be around 300 amino acids long. In addition, we used 2p4h as the baseline point and superimposed it onto the PDB structures listed in Table 1. The root-mean-square deviation (RMSD) ranged from 1.8 Å to 4.2 Å, indicating that all of the structures have similar folds.

**Table 1:** Threading analysis of 10 representative reductase containing sequences.

Reductase	Length	1sb8	1bkx	2hun	1db3	2b11	1kew	2pzk	1y1p	2p4h	1ek6	2c29	1udc	3gpi	2p5y	3enk	1r6d
		**341**	**339**	**329**	**335**	**294**	**361**	**310**	**342**	**310**	**346**	**324**	**338**	**273**	**311**	**340**	**320**

Peptaibol	422	{100}	{100}	{100}	{100}	-	-	-	C	C{100}	-	-	-	-	-	C	{100}
Lys2	476	C{100}	-	C{100}	{100}	-	-	-	C	C{100}	{100}	-	-	-	-	-	C{100}
Saframycin	427	{100}	{100}	{100}	-	-	{100}	{100}	C	C{100}	-	-	-	-	C	-	C{100}
Gramicidin	421	C{100}	{100}	{100}	{100}	-	{100}	{100}	-	C{100}	-	C	-	C	-	-	C
Myxochelin	414	{100}	C	{100}	{100}	C	C	{100}	C	C{100}	-	C	{100}	-	-	-	-
Lyngbyatoxin	416	C{100}	{100}	{100}	{100}	{100}	{100}	-	C	C{100}	-	C	-	-	-	-	C{100}
Myxalamid	407	C{100}	C{100}	C	{100}	{100}	-	C	-	C	C	-	C	-	C	C	{100}
BT peptide	462	{100}	{100}	{100}	-	{100}	{100}	-	-	C{100}	{100}	-	-	-	{100}	-	C{100}
Glycopeptidolipid	358	{100}	H{100}	{100}	-	-	{100}	H	-	C{100}	-	-	-	-	-	-	C
Nostocyclopeptide	389	{100}	C	-	{100}	{100}	-	C	C	C{100}	-	-	C	C	-	-	{100}

*The PDB IDs are listed for the structures that were aligned with these sequences during fold recognition analysis using the GenTHREADER and PHYRE servers. The results from GenTHREADER corresponding to confidence levels of CERTAIN and HIGH are labeled as C and H respectively. Similarly, PHYRE results corresponding to a precision level of 100% are labeled as {100}. (-) indicates the absence of a particular fold.

### Database search for multidomain proteins containing R domains

Next, we performed a BLAST search for 10 characterized proteins against the nr database to identify multidomain proteins containing the R domain. We eliminated single-domain proteins from the search results because our query was a multidomain protein and the cutoff amino acids length was ≥ 1000. Table 2 shows the program outputs categorized based on their classification in the NCBI database. This result showed that 57% of the R domains are present in NRPS and PKS gene clusters as multidomains, 31% were hypothetical and putative, and 12% were from other sources. Among the 252 multidomain proteins (Additional file 1), 226 proteins (92%) contained putative R domains at their extreme C-termini end, which might be involved in the product termination, while a stretch of 100-120 amino acids was present adjacent to the putative R domain at the C-terminal in the remaining 26 proteins. We collected these 26 protein sequences from the NCBI protein database and analyzed them by CDD (Additional file 2). The results revealed that these regions had short dehydrogenase (SD) domains with partial alignment, and that the *e-value *cutoff was less than the standard cutoff. These findings suggest that no other domain flanks the R domain. An earlier report suggested that the chemical structure of gramicidin A shows a four-electron reduction of the respective thioester, and that the second NADPH-dependent dehydrogenase LgrE, which is encoded by 3 kb upstream of the gene, is required to reduce an aldehyde intermediate into a primary alcohol product [47]. However, our analysis clearly showed that there is no second reductive domain like LgrE.

**Table 2:** Identification of multidomain proteins containing the R domain

Category	Total number of proteins
NRPS	118
PKS	27
Hypothetical	59
Unnamed protein	19
NAD-dependent epimerase	5
NRPS-PKS	7
Fatty acyl synthetase	5
Antibiotic synthetase	2
Others	10
	

Total	252

*A total of 252 multidomain proteins containing R domain were identified. The categorization is based on the NCBI database. The R domain is present in the NRPS and PKS system more often than in other systems.

### Homology modeling of the R domain

The R domain sequence has a low sequence identity (~20.5%) with VR, for which the 3D structure is available in the PDB (Pdb code: 2p4h). Although this identity level is not enough for homology modeling, its high similarity with VR (~44%), as suggested by our threading analysis, enabled us to model the R domain. The secondary structure of the R domain primary sequence predicted by the PHD server is shown in Additional file 3. The protein sequence features alternating α helices and β strands. The α helices account for 31.5% of the residues, while the β strands account for 16.5%. Specifically, the α helices are located at residues 13-26, 40-51, 66-69, 87-94, 122-133, 179-190, 219-227, 253-260, 280-289 and 300-312. The results predicted by MOE were similar to those predicted by the PHD server. The major difference among the predictions of PHD and MOE was that the MOE showed additional two strands (53-55 and 62-64) and a helix (146-149).

**Figure 3:**
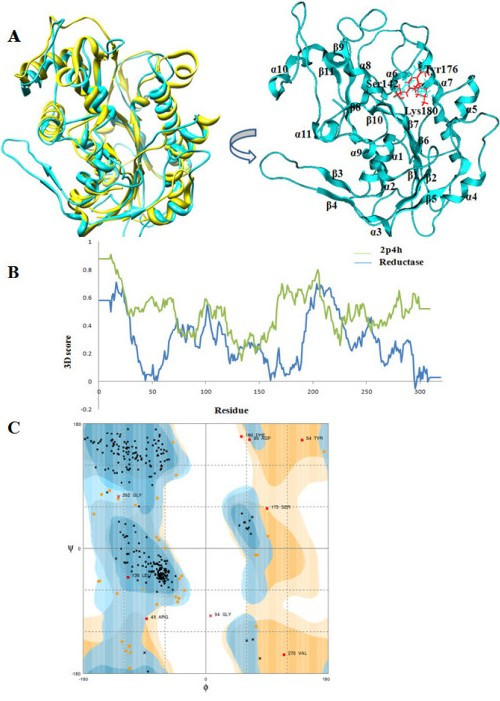
**Evaluation of the reductase domain model**. (A) The superposition of the modeled R domain (colored in cyan) with the crystal structure of VR (colored in yellow). The reductase characterizes the SYK triad for catalytic function (left). The modeled structure containing Ser142, Tyr176 and Lys180 is highlighted in cyan. The red residues are the corresponding residues of VR (right). (B) 3D structure profile for the modeled R domain. The structures of the template (2p4h) and the R domain are shown in green and blue color, respectively. (C) The Ramachandran plot of modeled reductase refined by MD simulation. A total of 270 residues (84.6%) fall in the most favored regions (cyan), while 38 residues (11.8%) fall in additional allowed regions (orange) and 12 residues (3.7%) fall in the additional allowed regions (pale orange). No residues fall in the additionally allowed region.

The alignment of the primary structure of the R domain along with its template, 2p4h, was used to construct the model (Figure 2B). Ten structures with different side chain conformations were obtained from the MOE-Homology model. These initial modeled structures were minimized by the coarse minimization procedure. The best energetic model with the highest packing score from these 10 models was used for further analysis. The final model of the R domain is superimposed with the template (2p4h) as shown in Figure 3A (left). The R domain was characterized as a non-metallo-oxidoreductase and contained the conserved Ser, Tyr and Lys (SYK) catalytic triad, which encompasses a Rossmann fold necessary for NADPH binding. It has been proposed that Tyr hydroxyl is the proton donor involved in the electrophilic attack on the substrate carbonyl in a reduction reaction. Lys facilitates the proton transfer from the hydroxyl oxygen of Tyr to the substrate. Ser participates in catalysis by stabilizing the reaction intermediates [52]. A comparison of our 3D model of the R domain with its template VR revealed several interesting structural differences. Two β strands (β3 and β11) and two helices (α7 and α11) are lost in the R domain, while the rest of the regions are identical with an RMSD (Cα coordinates) value of 2.34 Å (Figure 3A (left)).

### Evaluation of the reductase domain model

The final substrate-free model of the R domain was examined for the distribution of Φ and Ψ angles using Ramachandran plots generated within the MOE. The evaluation indicated that only 4 of 320 residues (1.25%) in the R domain are present in the outlying region, and that these residues were located far away from the active site of the model. Therefore, no further refinement of the model was required. The 3D structure profiles of both the R domain and 2p4h produced by the verify 3D structure evaluation server are shown in Figure 3B. The scores for nearly all residues in peptaibol were within reasonable regions. All of the above tests demonstrate that there is no structurally unreasonable region in the modeled 3D structure of the R domain and that the structure is satisfactory.

### Structural refinement and stability evaluation

**Figure 4:**
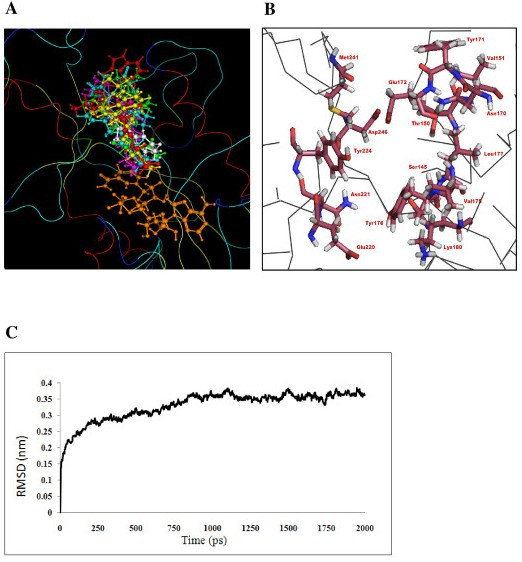
**Structural refinement**. (A) Spatial similarities of the VR and reductase domain binding sites. Vestitone (white line ball-and-stick) is superimposed on the R domain using the VR-vestitone docking coordinates. Energetically optimal conformations for Alamethicin (cyan, ball-and-stick), Trichotoxin (green, ball-and-stick), Antiamoebin I (magenta, ball-and-stick), Chrysopermin (yellow, ball-and-stick), Gramicidin (red, ball-and-stick) and NADPH (orange, ball-and-stick) predicted by MOE-Dock 2008.10 are pictured. For a given ligand, the result of each docking simulation is represented by a single chemical structure. (B) The putative substrate-binding pockets in the modeled structure of the R domain. Some amino acid residues in the binding pockets of the R domain are labeled and shown as a brown colored stick model. (C) RMSD of the R domain backbone atoms during 2-ns MD simulation.

The modeled reductase structure was refined and assessed for stability using MD simulation. The MD simulation is useful for identification of potential problems that may lead to instability of the modeled structure. After minimization and equilibration (~360 ps), 2-ns MD simulation was performed and data were collected for analysis. The potential energy (E_pot_) of the R domain is shown in Additional file 4. The potential energy decreases gradually from the start and then fluctuates around a flat basal line after ~1200 ps. The flat basal line indicates that the system is energetically stable after ~1200 ps. The RMSD value of the Cα atom between the snapshots and the initial structure was around 2.7 Å, with small fluctuations after about 800 ps. These findings suggest that proteins remain stable after reaching equilibrium. The RMSD between the initial modeled structure and the final refined structure is around 2.1 Å. The RMSD from the starting structure for all backbone Cα atoms as a function of simulation time is shown in Figure 4C. The modeled structure of the R domain is stable with both Rossmann fold and C-terminal substrate binding. The refined structure was further evaluated by the Procheck program [53] and the results are shown in Figure 3C. Most (96.4%) residues were found to fall in favored and additionally allowed regions, while the remaining 3.6% of the residues fell within the generously allowed regions. Thus, it is expected that the structural quality of the R domain model is sufficient for use in the investigation of protein-ligand docking.

### Docking validation of known vestitone-VR complexes

To evaluate our docking simulation, the crystal structure of VR (pdb code: 2p4h) and its ligand, vestitone, were downloaded from Pubchem and used to conduct two different docking simulations. The dominating clusters from the MOE-Dock 2008.10 and ASEDock docking simulations were found to have the same binding orientation when compared with the original VR crystal structure reported by Shao *et al. *[26]. Because the VR crystal structure does not contain the ligand, vestitone, the authors docked the ligand in the active site, then minimized the energy and elucidated the reaction mechanism. However, the similarity between the present docked poses and the residues reported by Shao *et al. *[26] is that our docking protocol is able to reproduce their reported near-native VR-vestitone complex. Therefore, we believe that our protocol is reliable and we used it in the subsequent docking calculations.

### Linear substrate-reductase interaction examination

The T domain in the final module transfers the substrates to the adjacent R domain through its 4'-phospopantetheine arm. Because the focus of this study was to elucidate the reaction taking place inside the R domain, and since the entire T domain is not involved in the reaction process, we did not include the entire T domain in our docking, but only its phosphopantetheine arm mimic. The refined structure of the R domain was employed in a docking simulation (MOE-Dock 2008.10) with the linear substrates alamethicin, antiamoebin I, trichotoxin, chrysopermin and gramicidin (Figure 1C). These five linear substrates are close structural analogs; therefore, it is not surprising that these ligands can be found in the same binding site in the R domain. Considering that R domain docking of the linear substrates employed an unbiased approach entirely independent of VR docking of its vestitone substrate, the overlap of alamethicin, antiamoebin I, trichotoxin, chrysopermin and gramicidin present in the reductase binding sites with the vestitone site of the analogous region of the VR crystal structure was found to be remarkable (Figure 4A). The coincidence of the substrate binding pockets within the reductase and VR proteins, which are highly dissimilar in sequence that recognizes structurally dissimilar substrates, in part validates the present R domain model.

The pocket near the catalytic site, which is presumably a substrate-binding site, is formed by loop α6-α7, loop β7-β6, helices α6, α7 and α8, and strands β6, β9 and β11. The amino acids that play a pivotal role in substrate recognition and catalysis are Ser145, Thr150, Val151, Val155, Asn170, Tyr171, Glu172, Tyr176, Leu177, Lys180, Asn210, Asp220, Asn221, Tyr224, M241 and D246 (Figure 4B). A close-up view of the peptidyl thioester substrates in the binding site (Figure 4A) shows an extensive spatial overlap of the predicted best poses, despite the variety of docking methods employed. The bound conformation of the ligand present in the R domain suggests that the thioester oxygen atom of all of the substrates can form a strong hydrogen bond with the OH group of Tyr176. Apart from these common and important interactions for all ligands, the other following interactions are possible: (i) The N-terminal amine group of alpha-aminoisobutyric acid1 (AIB1) of chryospermin forms a H-bond with the COOH group of Glu172, (ii) Chryospermin establishes a favorable hydrophobic interaction with Leu177 and Met241, (iii) In alamethicin, the N-terminal amine group of AIB1 and the carbonyl backbone of Gln2 form a H-bond with the carbonyl backbone of Met241 and the carboxamide side chain of Asn221, respectively, (v) a hydrophobic interaction of alamethicin with Leu277, Met241 and Met244, (vi) In antiamoebin, the OH group of hydroxyl porline1 and the carbonyl backbone of pro3 form a H-bond with the carbonyl backbone of Gln242 and the carboxamide side chain of Asn221, (vii) The antiamoebin forms a favorable hydrophobic interaction with Val146, Leu147, Met241 and Met244, (viii) In gramicidin, the N-terminal amine group and heterocyclic NH group of Trp1 form a H-bond with the carbonyl backbone of Met244 and the OH group of Tyr171, respectively, (ix) The gramicidin moiety establishes a favorable hydrophobic interaction with Val146, Leu177, Leu225, Met241 and Val245, (x) In trichotoxin, the carboxamide side chain of Gln3 and the carbonyl backbone of Gln3 form a H-bond with the carboxamide side chains of Asn242 and Asn221, respectively, and (xi) A hydrophobic interaction of trichotoxin with Leu177, Met241 and Val245.

**Figure 5:**
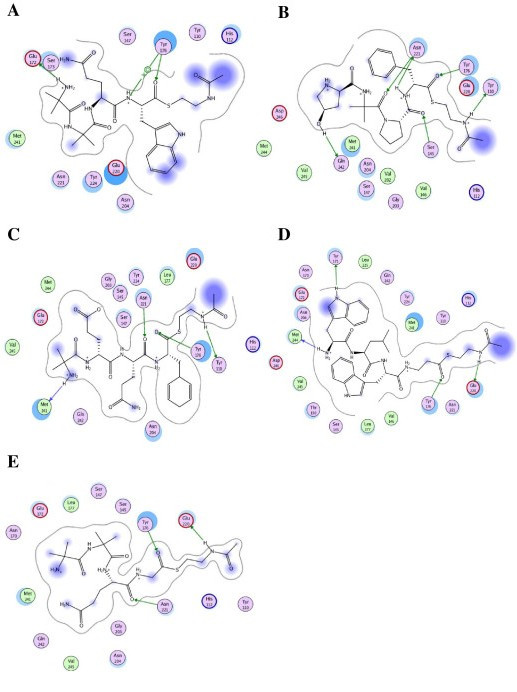
**Ligand interaction plot of the ASEDock-generated reductase Chrysopermin (panel A), Antiamoebin (panel B), Alamethicin (panel C), Gramicidin (panel D) and Trichotoxin (panel E)**. The plot depicts the 2D ("flattened") spatial arrangement of the ligand and the R domain with respect to key interactions. The proximity contour (dashed lines) and solvent exposed areas (solid purple spheres) of the ligand atoms are indicated as the polar (pink), hydrophobic (green), and solvent-exposed (light blue shadow) binding pocket amino acids. Acidic and basic residues are highlighted with red and blue halos, respectively.

The interaction energies (E_int_) for the substrates within the active site were calculated using the AMBER94 force field (Table 3). The interaction energies for the thioester carboxyl atoms were ≈ -38.911 kcal mol^-1^. As expected, the simulations suggested that the majority of the stabilization energy of the thioester oxygen atom in the receptor site is due to the hydrophobic interaction or van der Waals forces between them, with a lesser contribution from electrostatic interactions. In addition, the final docked complexes were further verified for the reactivity of the substrate using the Oprea topological rule [54]. All of the substrates within the active site of the R domain showed that thioester is reactive. To test the activity of the intermediate aldehyde, we deleted SNAc from all of the substrates in the docked complex, and then conducted energy minimization. The results showed that the aldehyde of the substrate is reactive. These findings indicate that the R domain is capable of conducting two steps in the reduction reaction. The MOE 2008.10 software was used to create ligand interaction plots for all of the substrates (Figure 5), which provided a clearer arrangement of putative key intermolecular interactions that aid in interpretation of the 3D juxtaposition of the ligand and R domain.

**Table 3:** The docking scores, interaction energies and binding affinity of the substrate obtained from the MOE-DOCK 2008.10 and the ASEDock simulation

Ligand	Molecular weight	ASEDock Score	MOE-Dock Score	E_int _(Kcal mol^-1^)	Affinity (pKi)	Distance between the protein (Tyr176 OH group) and the ligand thioester oxygen atom (Å)	Distance between the protein (Glu220 COOH group) and the ligand thiol group (Å)	Distance between C4 of NADPH and the thioester carbonyl carbon of the substrate (Å)
Alamethicin	612	-76.6624	-26.726	-43.399	14.776	2.33	3.52	4.13

Trichotoxin	477	-82.4472	-17.826	-32.511	12.817	2.59	3.35	3.35

Antiamoebin	564	-59.8066	-13.566	-34.452	14.467	2.11	3.65	3.71

Chryospermin	605	-77.2101	-25.093	-33.944	14.927	2.60	2.72	3.93

Gramicidin	677	-86.4472	-18.896	-50.249	17.066	3.06	3.70	3.86

Finally, we placed a NADPH in the R domain receptor site by superimposing 1Y1P (which has one NADPH molecule) onto the R domain, after which we minimized the energy (Figure 4A). NADPH consists of three fragments, adenine monophosphate (AMP), nicotinamide mononucleotide (NMN) and a phosphate (PO_4_) group. The distance between the C4 atom of the nicotinamide ring and the thioester carbonyl carbon atom of the substrates is shown in Table 3.

### Binding site comparison

The difference in the VR and reductase dockings and the catalytic activities were further evaluated by detailed analysis of the predicted binding sites using Computed Atlas of Surface Topography of proteins (CASTp) analysis [55,56]. Based on the results of this analysis, the binding site volumes without the various channels extending from the surface of the protein surface were estimated to be 2700 Å and 1733 Å for reductase and VR, respectively. The volume difference among the R domain and its template (VR) indicated a larger binding site for the R domain; therefore, the R domain can accept the substrate with the larger size.

## Discussion

The sequence-structure alignment plays a major role in providing reasonable and accurate structural predictions. However, in the case of low sequence identity, MOE has been employed to generate reasonable models for unknown 3D structures [57]. Because our sequence identity was low, we utilized MOE for homology modeling. The structural properties of the modeled R domain were further assessed for stability using MD simulation. The refined model generated during the MD simulation possessed satisfactory geometric parameters and was therefore used in the investigation of the ligand-protein interaction.

Docking calculations are generally used to predict ligand-protein conformations and help perform virtual screening of compounds present in chemical databases to identify therapeutic lead compounds [58-60]. Generally, the docking calculation is conducted when the location of the binding site is known or assumed. However, no docking calculations take full advantage of the pharmacologically characterized ligands when selecting the most feasible binding site. In the present study, two different docking approaches were employed to evaluate probable reductase binding sites by performing docking calculations using structurally linear, well characterized ligands as molecular probes.

Despite the different protein sequences and cognate substrate molecules, all ligand docking algorithms were located on the optimal binding pocket for alamethicin, trichotoxin, antiamoebin I, chrysopermin and gramicidin within the R domain model that was superimposable with the vestitone-VR docked structure (Figure 4A). The complex obtained by docking analysis had a critical hydrogen bond between the Tyr176-OH group of the reductase catalytic site and the C-terminal end of thioester oxygen of the substrate. In addition, the Glu220 carboxylic group of the R domain was located within the H-bond distance to the thiol group of the substrate. The protonation state of glutamic acid was stabilized through a hydrogen bonding. Considering the H-bond length, we found that Tyr176 was located at 2.6 Å from Glu220, maximizing the process of proton stabilization in Glu220 through a H-bond.

Further, our docking studies clearly indicated that the R domain was not influenced by the substrate amino acid chain. We have used five different substrates, whose last amino acids (L-configuration) of each substrate are Trp, Pro, Val, Phe and ethanolamine (ETA), respectively. Though they have different physiochemical properties, they bind exactly the same orientation with the R domain. Our findings are also supported by the study conducted by Schracke *et al. *[47], in which the substrate specificity of the R domain was evaluated by synthesizing Leu_12_-Trp_13_-Leu_14_-Leu_15_-Gly_16_-S-CoA and subsequently replacing Gly_16 _with Leu, Ala, Asp, Phe and Lys. They showed that R domain appears to be unspecific for the substrates.

Because the substrate lengths were ≈18-20 amino acids, we used the C-terminal end slots of 3, 4, 5 and 6 amino acids (residues) for the docking experiment to determine how many residues from the substrates could be accommodated in the active site of the R domain to catalyze the reaction mechanism. We found that the final 4 residues of the substrates bind in the active site, but that if there are greater than 4 residues the slot overlaps with the NADPH binding site. These results strongly imply that only the final four residues bind in the same catalytic site, while the remaining residues face away from the catalytic site and might be involved in the interaction with the side chain of the modeled R domain.

**Figure 6:**
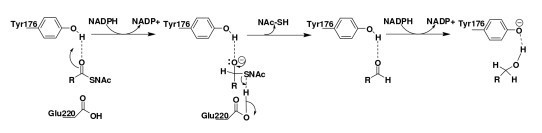
**Scheme of the reaction mechanism proposed for the R domain**. The two steps of the reduction reaction from the peptidyl thioester to the primary alcohol are shown, and the roles proposed for the key catalytic residues Tyr176 and Glu220 are indicated.

A BLAST search for R domains and our sequence analysis of primary alcohol products that possess a single R domain, such as myxalamid, lyngbyatoxin and peptaibols [9,15,40,43-48] clearly indicated that the reduction reaction took place in a single R domain, and that there is no other specific domain flanking the R domain. Based on this analysis, we suggest that the R domain catalyzes the reduction of acyl thioester into a primary alcohol product. Further, our docking studies identified two amino acids, Tyr176 and Glu220, which act as proton donors in the catalytic site of the R domain and are highly conserved among the characterized R domains (Figure 2A, marked by green and red asterisks). Therefore, we propose the following reduction reaction mechanism (Figure 6). During the first round of the reduction reaction, the substrate (e.g. alamethicin) binds with the R domain (active site), after which a hydrogen bond between the acyl thioester oxygen of the substrate and the OH group of Tyr176 (present in the active site) induces a partial carbonium ion characteristic in the thioester carbon. This facilitates the first hydride transfer from the C4 atom of NADPH to acyl thioester, forming a thioacetal and a NADP^+^. The hydrolysis of the C-S bond in the thioacetal forms an aldehyde bound to the Tyr176. Next, the phosphopantetheinyl sulfur (S^-^) accepts a proton from COOH group of Glu220 present in the active site, which results in the release of a phosphopantetheine SH group, at which point the second round of reduction can take place in the active site. However, before the second round of the reduction reaction, the previous NADP^+ ^will be released and the second NADPH will bind to the same Rossmann fold. During the second round of reduction, Tyr176, which is the proton donor of the carbonyl oxygen of the bound aldehyde, facilitates the second hydride transfer from NADPH, forming and releasing a primary alcohol. During this step, Tyr176 acts as a general base that is critically involved in the catalysis, which is known to be a common function of Tyr among all SDR family members [52]. It should be noted that this type of four electron reduction of peptidyl thioester into a corresponding alcohol product has only been briefly predicted by Read et al. [40] and Li et al [46]. Also, similar to the R domain, there are two other enzymes, 3-hydroxy-3-methylglutaryl-CoA reductase (HMGR) and UDP-glucose dehydrogenase (UDPDH), which can catalyze mechanistically related four-electron reduction (or oxidation) reactions [61,62]. Thus our study indicates that a terminal R domain may be responsible for the frequently used reductive release mechanism in NRPS systems.

However, a few studies have reported that free aldehyde products such as nostocyclopeptide, lys2 and saframycin are observed soon after the first round of the reduction reaction [49-51]. The basis for aldehyde release has not yet been explored and very few modeling reports are available to predict the reason for these findings. Although our modeling studies were not able to identify the mechanism by which the intermediate aldehyde can be released or remain in the same R domain and subsequently undergo a second round of reduction, we attempted to predict the reason for the observed behavior with the aid of threading analysis. Threading analysis showed that the alcohol-forming R domain (e.g., glycopeptidolipid) had 20.5% sequence identity with the selected template, 2p4h, while an aldehyde-forming R domain (e.g. saframycin) showed only 15% sequence identity with 2p4h. This prevents modeling of the aldehyde-forming R domain because modeling is generally only feasible when the sequence identity is >20% [16,63]. Additionally, the target-template homology between the aldehyde-forming R domain (e.g. saframycin) and 1r6d was < 17%, which also makes modeling of the aldehyde-forming R domain difficult. These findings indicate that there may be a structural difference between the alcohol- and aldehyde-forming R domains. The only limitation at present is the lack of a suitable template for modeling aldehyde-forming R domains to explore their function. Further studies in this direction are required to gain a complete understanding of the aldehyde-forming R domains.

## Conclusions

The results of receptor modeling and docking simulations demonstrate that, in NRPS proteins, the R domain always resided at the extreme C-terminal end that catalyzes the reduction of acyl thioester into its primary alcohol via an aldehyde intermediate through the four-electron reduction reaction. This four-electron reduction reaction was clearly described herein. Our study not only provides insight into the unknown TPS reaction mechanism, but also serves a starting point for identifying point mutations in active site residues that can enhance the selectivity and activity of the substrate.

## Authors' contributions

BM designed the experiments, conducted the analysis and wrote the manuscript. SKM helped in construction of the manuscript. GL and SC supervised the work. All authors read and approved the manuscript

## Supplementary Material

Additional file 1 **List of the proteins containing putative R domains**. Among the 252 multidomain proteins, 226 proteins (92%) contained putative R domains at their extreme C-termini end, which might be involved in the product termination, while a stretch of 100-120 amino acids was present adjacent to the putative R domain at the C-terminal in the remaining 26 proteins. Click here for file

Additional file 2 **Domain organization of sequences after R domain**. The sequences after R domain were collected and analyzed by CDD. The domain organizations of the entire length sequence are shown. Click here for file

Additional file 3 **Secondary structure prediction of TPS reductase**. The secondary structure was predicted by PHD. Among 320 residues, 101 (31.5%) residues are α helix, 52 (16.5%) are β sheet and 167 (52%) are random coil. Click here for file

Additional file 4 **The potential energy plot of 2 ns MD simulation**. During the simulation, the potential energy decreases from the start and then goes to a plateau after 1200 ps. The system intends to be stable. Click here for file
