# Supplementary material for: Molecular modeling of the reductase domain to elucidate the reaction mechanism of reduction of peptidyl thioester into its corresponding alcohol in non-ribosomal peptide synthetases
Source: BMC Struct Biol. 2010 Jan 12;10:1. doi: 10.1186/1472-6807-10-1 (PMC2835699; doi:10.1186/1472-6807-10-1)
Supplement: Additional file 2 — Domain organization of sequences after R domain. The sequences after R domain were collected and analyzed by CDD. The domain organizations of the entire length sequence are shown. [file 1472-6807-10-1-S2.DOC]

**Additional file 2**

| Protein accession number | Protein name | Organism | Domain organization |
| --- | --- | --- | --- |
| XP_391164.1 | Hypothetical protein FG10998.1 | *Gibberella zeae* | A-C-A-T-E-C-A-T-E-C-A-T-E-C-A-T-E-C-A-T-E-C-A-T-E-R-C-A |
| XP_958392.2 | Non ribosomal peptide synthetase | *Asperigillus fumigatus* | 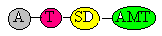 |
| XP_381856.1 | Hypothetical protein FG01680.1 | *Gibberella zeae* | 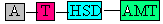 |
| XP_662922.1 | Hypothetical protein AN5318.2 | *Asperigillus nidulans* | 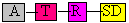 |
| XP_360747.1 | Hypothetical protein MG03290.4 | *Magnoporthe grisea* | 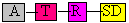 |
| XP_386683.1 | Hypothetical protein FG06507.1 | *Giberella zeae* | 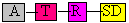 |
| AA049458.1 | Monomodular NRPS | *Leptosphaeria maculans* | 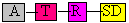 |
| BAE55724.1 | Unnamed protein product | *Asperigillus oryzae* | 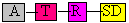 |
| AAX09992.1 | Non ribosomal peptide synthetase 10 | *Cochliobolus heterosptrophus* | 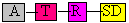 |
| XP_753630.1 | NRPS | *Asperigillus fumigatus* | 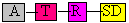 |
| AAX11423.1 | Nonribosomal peptide synthetase 10 | *Gibberella moniliformis* | 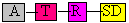 |
| Xp_759255.1 | Hypothetical protein UM03108.1 | *Ustilago maydis* | 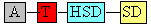 |
| XP_667412.1 | Hypothetical protein chro.30258 | *Cryptosporidium hominis* | 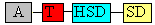 |
| *AAC99407.1 | Polyketide synthase | *Cryptosporidium parvum* | No specific hits |
| XP_625875.1 | Polyketide synthase | *Cryptosporidium parvum Iowa* | No specific hits |
| XP_659284.1 | Hypothetical protein AN1680.2 | *Asperigillus nidulans FGSC* | 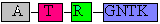 |
| XP_659284 | PREDICTED: Similar to ENSANGP00000010 | *Apis mellifera* | 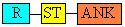 |
| CAD78888.1 | Polyketide biosynthesis | *Rhodopirellula baltica* | R-PKS-PKS-T-ACP-SD |
| XP_636393.1 | Hypothetical protein | *Dictyostelium discoideum* | 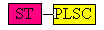 |
| YP_464376.1 | Long-chain-fatty-acid coA ligase | *Anaeroxomyxobacter dehalogenans* | 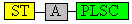 |
| EAL24834.1 | GA1396-PA | *Drosophilla pseudoobscura* | HSD-ST-ST-ST-ANK-ANK |
| EAS00429.1 | Male sterility protein | *Tetrahymena thermophila* | HSD-ST-ST-PLSC |
| XP_660007.1 | Hypothetical protein AN2403.1 | *Asperigillus nidulans FGSC* | 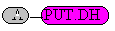 |
| **ABD72247.1 | Cell envelope proteinase A | *Streptococcus pyogenes* | 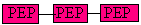 |

*Protein accession no indicates the same protein present again with different accession numbers. **Protein accession no indicates the same protein present more than two times with different accession numbers. The domain abbreviation is as follows: A-Adenylation, C-Condensation, T-Thiolation, R-Reductase, HSD-3β hydroxyl steroid dehydrogenase, PEP-Peptidase, PLSC-Phosphate acyl transferase, ST-Sterlity, GNTK-Gluconate kinase, ANK-Ankyrin, SD-Short chain dehydrogenase, PKS-Polyketide synthase, ADH- alcohol dehydrogenase, PUT.DH-Putative dehydrogenase.
