# Supplementary material for: Molecular modeling of the reductase domain to elucidate the reaction mechanism of reduction of peptidyl thioester into its corresponding alcohol in non-ribosomal peptide synthetases
Source: BMC Struct Biol. 2010 Jan 12;10:1. doi: 10.1186/1472-6807-10-1 (PMC2835699; doi:10.1186/1472-6807-10-1)
Supplement: Additional file 3 — Secondary structure prediction of TPS reductase. The secondary structure was predicted by PHD. Among 320 residues, 101 (31.5%) residues are α helix, 52 (16.5%) are β sheet and 167 (52%) are random coil. [file 1472-6807-10-1-S3.DOC]

**Additional file 3**

1--------0---------2---------3---------4---------5---------60

KEMEHVLLLGSTGFLGIHLLHELLQKTEATILCVIRAENDEAAMQRLRKKIDFYFTSQYS

EEEEEEE HHHHHHHHHHHHHH EEEEEEE HHHHHHHHHHH

---------7---------8---------9-------100-------110-------120

SSQIDEWFTRIQIIHGDITQANFGLEAKHYESLGAIVDTVIHTAALVKHYGHYEEFERAN

HHHH EEEEE HHHHHHHH EEEEEE

-------130-------140-------150-------160-------170-------180

VHGTQQVVTFCLNNKLPMHYVSTLSVSGTTVEEATELVEFTEKDFYVGQNYESNVYLRSK

HHHHHHHHHHHH EEEEEEEEE HH

-------190-------200-------210-------220-------230-------240

FEAEAVLVGGMENGLDARIYRVGNLTGRFQDGWFQENINENMFLLSKAFLELGGFDQEIM

HHHHHHHHHH EEEEEE HHHHHHHHH

-------250-------260-------270-------280-------290-------300

QGMVDLTPIDICAQAIIHIINSKGIEERVFHLQNPHLVTYDDMYRVFEGLGFSRRVQSRE

EEE HHHHHHHH EEE EEEEEE HHHHHHHHHH H

-------310---------

DVTRELDVMMSQGNEKLFL

HHHHHHHHHHHH
