# Supplementary figures and images for: Molecular modeling of the reductase domain to elucidate the reaction mechanism of reduction of peptidyl thioester into its corresponding alcohol in non-ribosomal peptide synthetases
Source: BMC Struct Biol. 2010 Jan 12;10:1. doi: 10.1186/1472-6807-10-1 (PMC2835699; doi:10.1186/1472-6807-10-1)

**Additional file 4**


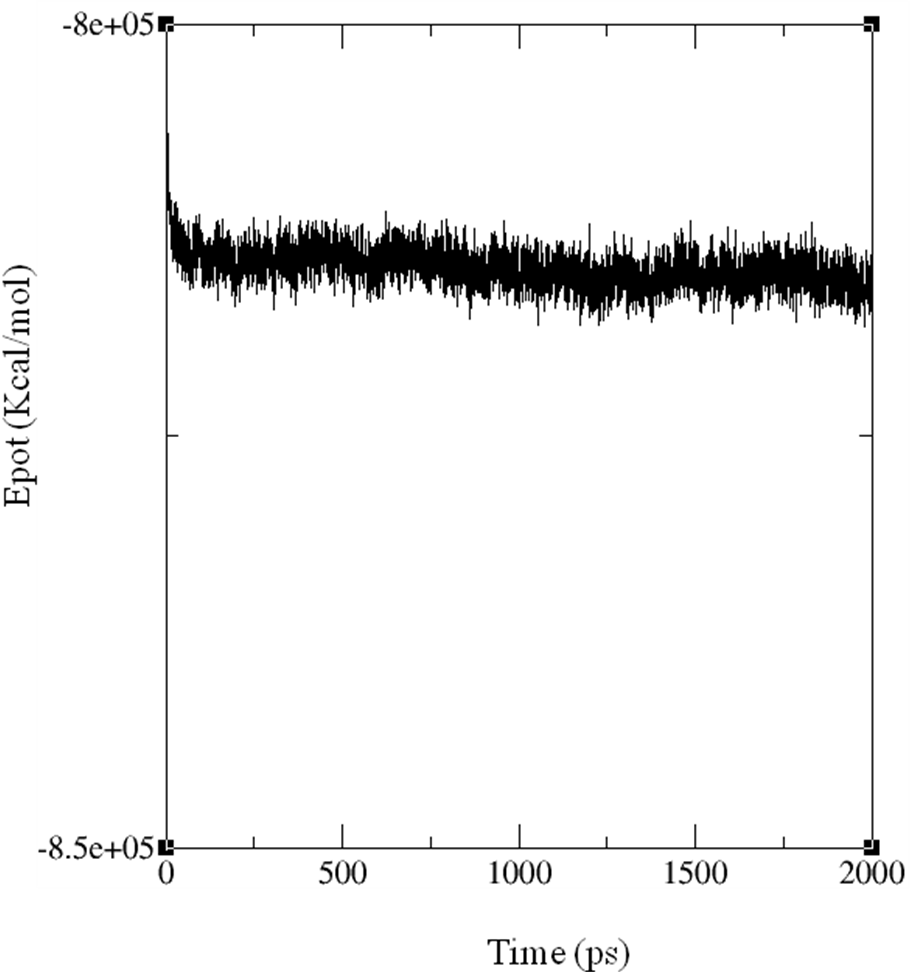

Supplement: Additional file 4 — The potential energy plot of 2 ns MD simulation. During the simulation, the potential energy decreases from the start and then goes to a plateau after 1200 ps. The system intends to be stable. [file 1472-6807-10-1-S4.DOC]
